# The Relationship Between Lipoprotein-Associated Phospholipase-A2 and Coronary Artery Aneurysm in Children With Kawasaki Disease

**DOI:** 10.3389/fped.2022.854079

**Published:** 2022-03-31

**Authors:** Zhenli Cheng, Haobo Weng, Jing Zhang, Qijian Yi

**Affiliations:** ^1^Department of Cardiovascular Medicine, National Clinical Research Center for Child Health and Disorders, Ministry of Education Key Laboratory of Child Development and Disorders, China International Science and Technology Cooperation Base of Child Development and Critical Disorders, Children's Hospital of Chongqing Medical University, Chongqing, China; ^2^Chongqing Key Laboratory of Pediatrics, Chongqing, China

**Keywords:** Kawasaki disease, lipoprotein-associated phospholipase A2, coronary artery aneurysm, children, pediatric cardiology

## Abstract

**Background:**

Coronary artery lesions including aneurysm, as the most severe complications of Kawasaki disease (KD), remain of great concern. Lipoprotein-associated phospholipase A2 (Lp-PLA2) is implicated in the regulation of inflammatory response and lipid metabolism. Since excessive inflammatory response and aberrant lipid metabolism have involved in the development of KD, we in this study sought to investigate the relationship between coronary artery aneurysm (CAA) and Lp-PLA2 and other blood parameters in children with KD.

**Methods:**

The participants included 71 KD patients, 63 healthy controls (HCs) and 51 febrile controls (FCs). KD patients were divided into KD-CAA (KD with CAA) group and KD-NCAA (KD without CAA) group. Serum Lp-PLA2 levels were measured using enzyme-linked immunosorbent assays. Other routine clinical parameters were also detected.

**Results:**

Serum Lp-PLA2 levels in KD group [4.83 μg/mL (3.95–6.77)] were significantly higher than those in HC [1.29 μg/mL (0.95–2.05)] and FC [1.74 μg/mL (1.18–2.74)] groups. KD-CAA group [5.56 μg/mL (4.55–22.01)] presented substantially higher serum Lp-PLA2 levels as compared with KD-NCAA group [4.64 μg/mL (2.60–5.55)]. In KD group, serum Lp-PLA2 level was positively related with erythrocyte sedimentation rate, the levels of leukocytes, platelets, albumin, creatine kinase-MB, and D-dimer, and the *Z-*scores of left main CA, right CA, left anterior descending CA, and left circumflex CA; and negatively related with mean corpuscular hemoglobin concentration and mean platelet volume. Moreover, receiver operating characteristic curves showed that Lp-PLA2 exhibited superior and moderate diagnostic performance for distinguishing KD patients from HC and FC ones, respectively, and possessed the potential ability to predict the occurrence of CAAs in KD.

**Conclusion:**

Lp-PLA2 may be related to KD and the formation of CAAs, and thus may serve as a potential diagnostic biomarker for KD.

## Background

Kawasaki disease (KD), also known as mucocutaneous lymph node syndrome, is an acute febrile pediatric disorder characterized by systemic and self-limited vasculitis in small-sized and especially medium-sized arteries (1). KD predominantly afflicts infants and children under age of 5 years. Its pathogenesis has been universally attributed to inflammatory cascade and aberrant immunoreaction. Coronary artery aneurysm (CAA) is recognized as the most important complication of KD and is a high-risk factor for myocardial ischemia, infarction, and even coronary artery rupture (2, 3). Although KD was initially reported by Dr. Tomisaku Kawasaki in 1967 (4), its specific pathogenesis remains obscure. Intravenous immunoglobulin (IVIG) still persists as the mainstay of KD treatment. However, KD in ~10–20% of its patients fail to response to IVIG treatment, and it is more likely to bring about CAAs (2, 5–8). KD has become the leading cause of pediatric acquired heart disease in the developed countries (2). It is urgent, therefore, to further uncover the potential pathogenesis of the development of KD and CAAs, and find more effective treatment strategies.

Lipoprotein-associated phospholipase A2 (Lp-PLA2) is a 45kDa calcium-independent hydrophobic protein that belongs to the phospholipase A2 superfamily (9). Lp-PLA2 is predominantly secreted by monocytes and macrophages, and mainly exists in combination with low-density and high-density lipoprotein particles in blood circulation (10, 11). It was initially identified as an enzyme capable of hydrolyzing platelet-activating factor and truncate phospholipids in the settings of inflammation and oxidant stress (12, 13), and it was deemed as a mediator of inflammation and lipid metabolism (14). Mounting studies have demonstrated recently that Lp-PLA2 is associated with coronary heart disease (15–17). At present, Lp-PLA2 has been applied as a potential biomarker for predicting the risk of coronary heart disease and ischemic stroke associated with atherosclerosis (15, 18). Certain researches have proved that KD is a kind of systematic vasculitis caused by excessive inflammatory response (2). Taken together, it seems that the dysregulation of lipid metabolism may be one of the potential pathogenic mechanisms of KD (19, 20). We therefore speculated that Lp-PLA2 might be related to KD and its complications. Thus, we in this study aimed to investigate the relationships between Lp-PLA2 and KD, and Lp-PLA2 and CAA.

## Methods

### Participants and Sample Collection

In this retrospective study, all the participants were enrolled from Children's Hospital of Chongqing Medical University (Chongqing, China). The diagnosis criteria of KD were rigorously based on the guidelines proposed by Japanese Circulation Society Joint Working Group (21). KD patients with inflammatory diseases, immune diseases, metabolic diseases, hematological diseases or other heart diseases were excluded. A total of 71 children with KD were recruited (male: 46, female: 25, age: 29.44 ± 22.52 months) in KD group. We also recruited 63 healthy children (male: 41, female: 22, age: 30.33 ± 11.59 months) from health checkup for HC (healthy control) group, and 51 children (male: 29, female: 22, age: 28.05 ± 14.68 months) with acute febrile disease for FC (febrile control) group (Supplementary Table S1). FCs included patients with bronchopneumonia, infectious diarrhea, urinary tract infection, sepsis, hand-foot-mouth disease, or infectious mononucleosis.

Echocardiography was performed 1 day before treatment with intravenous immunoglobulin (IVIG) and anticoagulants in KD children. All the cardiac ultrasound examinations were performed by the expert physicians who were unblinded to the clinical information. Patients with *Z-*score > 2.5 were allocated into KD-CAA (KD patients with CAAs) group (*n* = 33), and those with *Z-*score <2.5 were into KD-NCAA (KD patients without CAAs) group (*n* = 38) (22).

All venous blood samples were collected before treatment with IVIG and anticoagulants. The samples were centrifuged at 3,000 rpm for 10 min to separate serum and red blood cells, and then stored at −80°C. Same sampling procedure was performed in healthy controls and febrile controls.

This study was approved by the Ethics Committee of Children's Hospital, Chongqing Medical University (Chongqing, China), and informed consents were obtained from the parents or guardians. All methods were carried out in accordance with relevant guidelines and regulations addressed in the Declaration of Helsinki.

### Measurement of Serum Levels of Lp-PLA2 and Routine Blood Parameters

Serum levels of Lp-PLA2 were determined by quantitative enzyme-linked immunosorbent assay as per the manufacturer's instructions (Cloud-Clone Corp USCN Life Science, Wuhan, China). Routine blood parameters were also measured in the blood samples, including white blood cell (WBC) count, percentage of neutrophils, percentage of leukomonocytes, red blood cell count, hemoglobin, mean corpuscular volume, mean corpuscular hemoglobin concentration (MCHC), erythrocyte sedimentation rate (ESR), procalcitonin, aspartate aminotransferase, alanine aminotransferase, albumin (ALB), creatine kinase-MB (CK-MB), platelet (PLT) count, C-reactive protein, *Z-*score of left main coronary artery (LMCA), *Z-*score of right coronary artery (RCA), *Z-*score of left anterior descending coronary artery (LAD), *Z-*score of left circumflex coronary artery (LCX), as well as blood coagulation parameters such as prothrombin time, activated partial thromboplastin time, fibrinogen, thrombin time and D-dimer (DD).

### Statistical Analysis

All statistical analyses were performed using the SPSS software for Windows (Version 25.0, SPSS, Chicago, IL, USA). Student's *t-*test was used for the data simulated from a normal distribution, and the nonparametric Mann-Whitney U test was applied to analyze the data that failed the normality assumption. Chi-square test was used for comparisons of frequencies between groups. Spearman's rank correlation was used to measure the correlation between serum Lp-PLA2 level and other clinical parameters. Receiver operating characteristic (ROC) curves were plotted and areas under the curves (AUC) were calculated to evaluate the diagnostic performance of Lp-PLA2 for KD. All values were shown as mean ± standard deviation, median (P25-P75) or number (percentage). Two-sided *p*-values < 0.05 were considered statistically significant.

## Results

### Characterization of Serum Lp-PLA2 and Routine Clinical Parameters in All Groups

**Figure 1:**
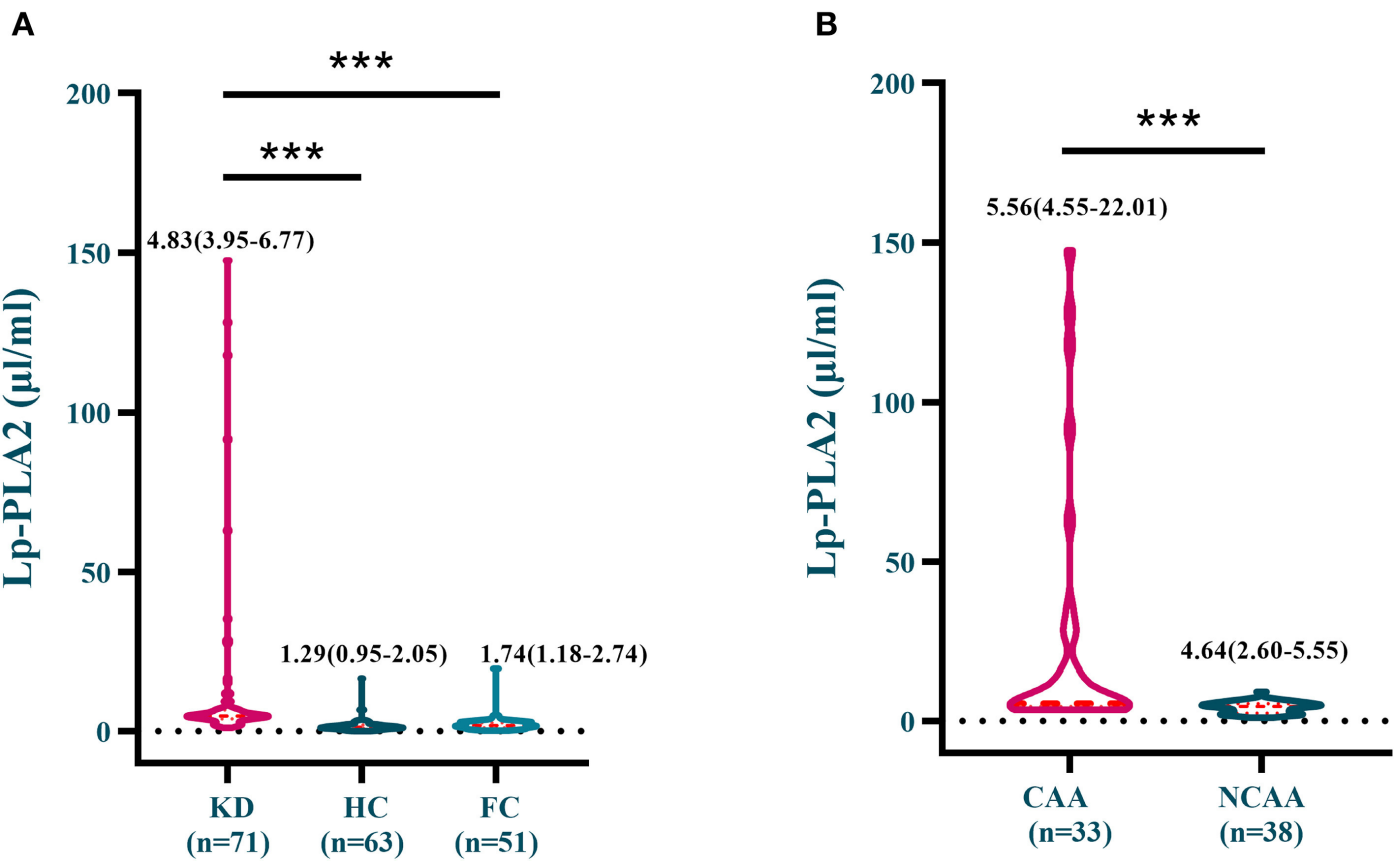
**(A)** Serum Lp-PLA2 levels in the KD, the HC and the FC groups; **(B)** Serum Lp-PLA2 levels in the KD-CAA and the KD-NCAA groups. ***p < 0.0001.

As presented in Figure 1, serum Lp-PLA2 levels in KD group [4.83 μg/mL (3.95–6.77)] were significantly higher than those in HC [1.29 μg/mL (2.05)] and FC groups [1.74 μg/mL (1.18–2.74)]. KD-CAA group [5.56 μg/mL (4.55–22.01)] showed substantially higher Lp-PLA2 level in contrast to KD-NCAA group [4.64 μg/mL (2.60–5.55)].

There was no significant difference in percentage of neutrophils, percentage of lymphocytes, red blood cell count, mean corpuscular volume, ESR, and the levels of hemoglobin, procalcitonin, aspartate aminotransferase, alanine aminotransferase, ALB, CK-MB, and C-reactive protein between KD-CAA and KD-NCAA groups. However, KD-CAA group presented significantly higher WBC and PLT levels and lower MCHC by contrast with KD-NCAA group (Table 1).

**Table 1:** General laboratory variables and serum Lp-PLA2 levels in KD-CAA and KD-NCAA groups.

	**KD-CAAs (*n =* 33)**	**KD-NCAAs (*n =* 38)**	***p-*value**
WBC (10^3^/μl)	16.56 ± 4.85	13.33 ± 3.89	0.003^*^
N%	0.63 ± 0.17	0.67 ± 0.12	0.207
L%	0.31 ± 0.15	0.26 ± 0.11	0.158
RBC (10^3^/μl)	4.12 ± 0.43	4.06 ± 0.29	0.573
Hb (g/l)	104.60 ± 12.07	108.60 ± 6.77	0.093
MCV (fl)	77.78 ± 6.21	80.01 ± 3.44	0.086
MCHC (g/l)	326.10 ± 12.98	333.10 ± 9.40	0.019^*^
ESR (mm/h)	65.21 ± 28.35	64.50 ± 27.56	0.924
PCT (%)	0.39 (0.15–2.15)	0.26 (0.11–0.68)	0.259
AST (U/l)	26.80 (24.55–34.30)	27.15 (23.85–33.15)	0.654
ALT (U/l)	22.65 (13.55–62.18)	22.75 (14.60–40.55)	0.993
ALB (g/l)	36.30 ± 4.48	36.75 ± 4.10	0.669
CK-MB (U/l)	0.87 ± 0.59	1.09 ± 0.61	0.150
PLT (10^9^/l)	456.30 ± 179.40	369.90 ± 110.10	0.018^*^
CRP (mg/dl)	59.77 ± 41.88	52.00 ± 38.51	0.436
Lp-PLA2 (μg/ml)	5.56 (4.55–22.01)	4.64 (2.60–5.55)	<0.001^*^

Lp-PLA2, lipoprotein-associated phospholipase A2; KD, Kawasaki disease; CAAs, coronary artery aneurysms; NCAAs, non-CAAs; WBC, white blood cell counts; N%, percentage of neutrophils; L%, percentage of leukomonocytes; RBC, red blood cell counts; Hb, hemoglobin; MCV, mean corpuscular volume; MCHC, mean corpuscular hemoglobin concentration; ESR, erythrocyte sedimentation rate; PCT, procalcitonin; AST, aspartate aminotransferase; ALT, alanine aminotransferase; ALB, Albumin; CK-MB, creatine kinase-MB; PLT, platelet counts; CRP, C-reactive protein; ^*^P <0.05.

As shown in Tables 2–4, serum Lp-PLA2 levels, in KD group, were positively related with ESR, the levels of WBC, ALB, CK-MB, and the *Z-*scores of LMCA, RCA, LAD and LCX, and negatively related with MCHC. Likewise, KD-CAA group saw a similar trend in terms of those parameters, and in addition, its serum Lp-PLA2 levels were strongly related to WBC level and the *Z-*scores of aforementioned branches of coronary arteries.

**Table 2:** Correlations between serum Lp-PLA2 levels and general laboratory variables in KD group.

	**Lp-PLA2**	
	***r***	***p-*value**
WBC (10^3^/μl)	0.427	<0.001^*^
N%	0.126	0.307
L%	−0.116	0.347
RBC (10^3^/μl)	−0.124	0.312
Hb (g/l)	−0.159	0.196
MCV (fl)	0.077	0.559
MCHC (g/l)	−0.267	0.040^*^
ESR (mm/h)	0.268	0.046^*^
PCT (%)	−0.093	0.467
AST (U/l)	−0.075	0.545
ALT (U/l)	0.095	0.440
ALB (g/l)	−0.241	0.047^*^
CK-MB (U/l)	−0.279	0.021^*^
CRP (mg/l)	0.088	0.489

Lp-PLA2, lipoprotein-associated phospholipase A2; WBC, white blood cell counts; N%, percentage of neutrophils; L%, percentage of leukomonocytes; RBC, red blood cell counts; Hb, hemoglobin; MCV, mean corpuscular volume; MCHC, mean corpuscular hemoglobin concentration; ESR, erythrocyte sedimentation rate; PCT, procalcitonin; AST, aspartate aminotransferase; ALT, alanine aminotransferase; ALB, Albumin; CK-MB, creatine kinase-MB; CRP, C-reactive protein; ^*^P <0.05.

**Table 3:** Correlations between serum Lp-PLA2 levels and general laboratory variables in KD-CAAs and KD-NCAAs groups.

	**KD-CAAs (*n =* 33)**		**KD-NCAAs (*n =* 38)**	
	**r**	***p-*value**	**r**	***p-*value**
WBC (10^3^/μl)	0.438	0.012^*^	0.363	0.030^*^
N%	0.233	0.200	0.383	0.021^*^
L%	−0.235	0.195	−0.377	0.024^*^
RBC (10^3^/μl)	−0.191	0.296	−0.275	0.104
Hb (g/l)	−0.107	0.558	−0.034	0.842
MCV (fl)	0.206	0.293	0.180	0.325
MCHC (g/l)	−0.229	0.240	0.344	0.054
ESR (mm/h)	0.389	0.041	0.086	0.665
PCT (%)	−0.216	0.242	0.047	0.797
AST (U/l)	−0.166	0.365	0.295	0.080
ALT (U/l)	0.078	0.671	0.230	0.177
ALB (g/l)	−0.330	0.065	−0.060	0.727
CK-MB (U/L)	−0.357	0.045^*^	0.134	0.435
CRP (mg/L)	0.072	0.711	0.054	0.758

Lp-PLA2, lipoprotein-associated phospholipase A2; KD, Kawasaki disease; CAAs, coronary artery aneurysms; NCAAs, non-CAAs; WBC, white blood cell counts; N%, percentage of neutrophils; L%, percentage of leukomonocytes; RBC, red blood cell counts; Hb, hemoglobin; MCV, mean corpuscular volume; MCHC, mean corpuscular hemoglobin concentration; ESR, erythrocyte sedimentation rate; PCT, procalcitonin; AST, aspartate aminotransferase; ALT, alanine aminotransferase; ALB, Albumin; CK-MB, creatine kinase-MB; CRP, C-reactive protein; ^*^P < 0.05.

**Table 4:** Correlations between serum Lp-PLA2 levels and *Z-*value.

	**Lp-PLA2**					
	**KD (*n =* 71)**		**KD-CAA (*n =* 33)**		**KD-NCAA (*n =* 38)**	
	***r***	***p-*value**	***r***	***p-*value**	***r***	***p-*value**
*z-*value of LMCA	0.640	<0.001^*^	0.649	<0.001^*^	−0.274	0.218
*z-*value of RCA	0.666	<0.001^*^	0.758	<0.001^*^	−0.107	0.652
*z-*value of LAD	0.482	0.002^*^	0.504	0.012^*^	0.107	0.692
*z-*value of LCX	0.578	<0.001^*^	0.582	0.001^*^	0.136	0.546

Lp-PLA2, lipoprotein-associated phospholipase A2; KD, Kawasaki disease; LMCA, left main coronary artery; RCA, right coronary artery; LAD, left anterior descending coronary artery; LCX, left circumflex coronary artery; ^*^P < 0.05.

With regards to blood coagulation, serum Lp-PLA2 levels were positively related with PLT and DD levels in KD and KD-CAA groups. Moreover, in KD group, serum Lp-PLA2 levels were negatively related with MPV (Table 5).

**Table 5:** Correlations between serum Lp-PLA2 levels and blood coagulation parameters.

	**LP-PLA2**					
	**KD (*n =* 71)**		**KD-CAA (*n =* 33)**		**KD-NCAA (*n =* 38)**	
	***r***	***p-*value**	***r***	***p-*value**	***r***	***p-*value**
PLT (10^3^/μl)	0.512	<0.001^*^	0.549	0.001^*^	−0.090	0.602
MPV (fl)	−0.266	0.042^*^	−0.352	0.072	−0.210	0.250
PDW (fl)	−0.253	0.053	−0.346	0.077	−0.244	0.179
PT (s)	0.144	0.249	0.111	0.551	0.170	0.328
APTT (s)	0.070	0.575	0.078	0.678	0.239	0.167
FIB (g/l)	0.013	0.919	0.110	0.556	0.324	0.057
TT (s)	−0.027	0.830	−0.131	0.481	−0.127	0.466
DD (mg/l)	0.501	0.015^*^	0.547	0.053	0.564	0.089

Lp-PLA2, lipoprotein-associated phospholipase A2; KD, Kawasaki disease; CAAs, coronary artery aneurysms; NCAAs, non-CAAs; PLT, platelet counts; MPV, mean platelet volume; PDW, platelet distribution width; PT, prothrombin time; APTT, activated partial thromboplastin time; FIB, fibrinogen; TT, thrombin time; DD, D-dimer; ^*^P < 0.05.

### Diagnostic Performance of Lp-PLA2 for KD

**Figure 2:**
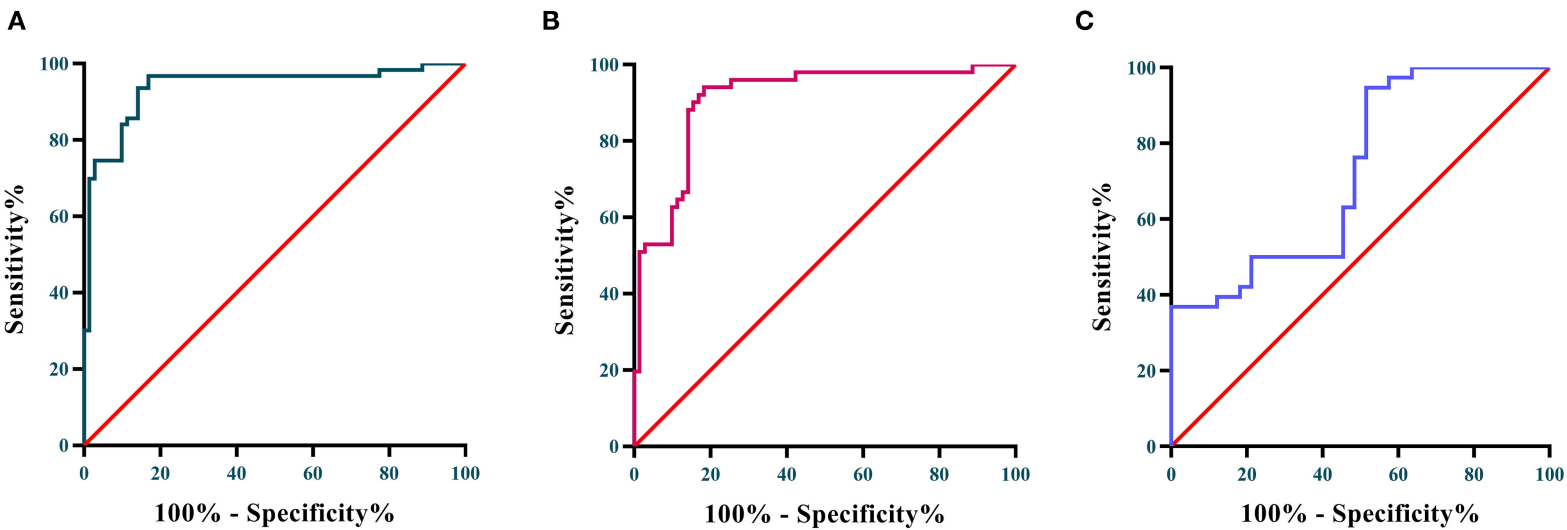
**(A)** ROC curves of Lp-PLA2 for distinguishing KD patients from HCs; **(B)** ROC curve of Lp-PLA2 for distinguishing KD from FCs; **(C)** ROC curve of Lp-PLA2 for predicting the occurrence of CAA.

The diagnostic performance of Lp-PLA2 between KD patients and HCs, and KD patients & FCs were verified using receiver operating characteristic (ROC) analysis. As shown in Figures 2A,B, Lp-PLA2 exhibited superior diagnostic performance in distinguishing the KD patients from HCs. The AUC value was 0.939 (95% confidence interval 0.895–0.983, *P* < 0.0001), and the cut-off value was 1.743 μg/mL with sensitivity and specificity ratios of 69.84% and 98.59%, respectively. In addition, Lp-PLA2 possessed capacity to distinguish the KD patients from FCs. The ROC analysis showed that the AUC value was 0.910 (95% confidence interval 0.856–0.964, *P* < 0.0001), and the cut-off value was 1.756 μg/mL with sensitivity and specificity ratios of 50.98 and 98.59%, respectively. As presented in Figure 2C, Lp-PLA2 also had moderate diagnostic performance for predicting CAAs in the KD patients. The AUC value was 0.725 (95% confidence interval 0.606–0.844, *P* = 0.001), and the cut-off value was 3.700 μg/mL with sensitivity and specificity ratios of 38.64 and 96.97%, respectively.

## Discussion

Lp-PLA2, conventionally known as platelet-activating factor acetyl hydrolase, is a unique member of the A2 phospholipase superfamily (23). It is synthesized mainly by macrophages and monocytes and binds to LDL and HDL particles in blood circulation (10). Previous studies have demonstrated the strong association of Lp-PLA2 with some inflammation-related cardiovascular diseases (14, 18, 24), and its involvement in the regulation of inflammation and lipid metabolism (25). KD is recognized as a severe inflammatory disorder associated with systemic vasculitis of unclear etiology. Excessive inflammation due to the presence of certain inflammatory mediators has been universally perceived as the contributor to KD development (2). Moreover, recent studies have found that adipocytokines like resistin and adiponectin were aberrantly expressed in the acute phase of KD (19, 26). This phenomenon indicates that abnormal lipid metabolism may also contribute to the formation of KD.

In the current study, we found that serum Lp-PLA2 levels were significantly increased in the KD patients. In particular, KD patients complicated with CAAs had substantial higher serum Lp-PLA2 levels. Moreover, we performed correlation analyses and found serum Lp-PLA2 levels were positively related to ESR, the levels of WBC, ALB, CK-MB, PLT, and DD, and the *Z-*scores of LMCA, RCA, LAD, and LCX, but negatively associated with MCHC and MPV. Furthermore, we found Lp-PLA2 exhibited superior diagnostic performance for distinguishing KD patients from healthy and febrile children, and also possessed the capacity to predict the occurrence of CAAs.

Lp-PLA2 is initially identified as an enzyme that contributes to degradation and inactivation of platelet-activating factor. It is also responsible for catalyzing hydrolysis of phospholipids within oxidized LDL molecules at the sn-2 position, which could contribute to the generation of pro-atherogenic and pro-inflammatory mediators, lysophosphatidylcholine and oxidized non-esterified fatty acids, and thus lead to the dysfunction of endothelial cells and smooth muscle cells (9, 12). Researches have shown that the damage to vascular endothelial cells and smooth muscle cells caused by excessive inflammatory response plays a critical role in the development of KD (1). This study demonstrated that serum Lp-PLA2 levels were significantly elevated in the KD patients, and were positively related to inflammation-related clinical parameters such as WBC and ESR. It indicated that Lp-PLA2 was more likely to be a pathogenic factor in KD. Besides, Lp-PLA2 also has an anti-inflammatory and anti-atherogenic role. Introduction of Lp-PLA2 gene into apoE-deficient mice would lead to a reduction in the mean arterial wall thickness and less spontaneous atherosclerosis (11). This phenomenon is due to the failure of mouse LDL to bind to Lp-PLA2 and thus the elevation of serum HDL-associated Lp-PLA2. Lp-PLA2 exerts its anti- or pro-inflammatory, and anti- or pro-atherogenic effects depending on its binding to high-density or low-density lipoproteins. Therefore, further studies are required to demonstrate whether Lp-PLA2 is involved in KD as a pathogenic or protective factor. Moreover, previous studies found that adipocytokines was significantly up-regulated in the KD patients (19, 26), and resistin could aggravate coronary artery lesions via nuclear factor-kB signaling pathway in KD (20). Lp-PLA2 and resistin, mainly expressed by macrophages, are both coincidentally involved in some immune-related or lipid-related diseases (27), such as rheumatoid arthritis (27, 28), atherosclerosis (23, 29), obesity, and diabetes (30). There might be an association between Lp-PLA2 and resistin. Based on the results and these evidence, we could speculate that Lp-PLA2 might be related to KD, However, further studies are required to verify the findings.

CAA is the most important complication of KD, and ~20% KD patients would develop CAAs even after standard treatment (infusion of IVIG) (2, 3). Therefore, novel therapy targets are critically required for improving the prognosis of KD patients. In this study, we found that serum Lp-PLA2 levels in KD-CAA group were significantly higher than those in KD-NCAA group. Correlation analysis showed that serum Lp-PLA2 levels were positively related with *Z-*scores of LMCA, RCA, LAD, and LCX. These results implied that Lp-PLA2 might strongly related to the formation of CAAs. Numerous studies have demonstrated that Lp-PLA2 is related to coronary heart disease. In atherosclerosis, Lp-PLA2 and its hydrolyzed products, viz, lysophosphatidylcholine, are abundant in the necrotic cores and thin fibrous caps of the lesions (10). Lp-PLA2 would be released throughout the body from macrophages and monocytes, possibly under the stimulation of certain proinflammatory cytokines, and is mainly carried by LDL in blood circulation (11). The LDL and Lp-PLA2 complexes penetrate the artery wall and locate in the subendothelial space. Additionally, in a deleterious feed-forward manner, Lp-PLA2 in the pathological areas could recruit macrophages, T-cells and mast cells, which might cause further release of Lp-PLA2 (10, 23). Certain CD163-positive macrophages were observed in the lesions of vessels in the acute phase of KD (31). It is relatively straightforward to envisage a scenario that Lp-PLA2 was released by macrophages under inflammation and accumulated in the artery wall; subsequently, macrophages would be recruited by Lp-PLA2 in the artery wall and might further release Lp-PLA2 and other inflammatory cytokines, and eventually lead to the damage to and dysfunction of endothelial cells and smooth muscle cells that contribute to CAAs.

Clinical researches have shown that high serum Lp-PLA2 levels were more likely to have a coronary event in atherosclerosis, and Lp-PLA2 was used as a biomarker for predicting a patient's risk for coronary heart disease and ischemic stroke associated with atherosclerosis (24). In this study, we analyzed the diagnostic performance of Lp-PLA2 using ROC analysis, along with the calculation of AUC values. We found Lp-PLA2 had superior diagnostic performance for distinguishing the KD patients from healthy children, with an AUC value of 0.921; and the cut-off value was 1.688 μg/mL with sensitivity and specificity ratios of 61.90 and 98.59%, respectively. In addition, Lp-PLA2 had moderate diagnostic performance for differentiating the KD patients from febrile children. Reliable predictive biomarkers for CAAs are still scarce (32) although there are various biomarkers for the diagnosis of KD and the prediction of IVIG-resistance (33, 34). In this study, we found that serum Lp-PLA2 had modest diagnostic potential for predicting the occurrence of CAAs, and the AUC value was 0.725 (95% confidence interval 0.606–0.844, *P* = 0.001), with sensitivity and specificity ratios of 38.64% and 96.97% respectively under the most appropriated cut-off value of 3.700 μg/mL. These findings might facilitate the early diagnosis and management of CAAs in KD patients.

## Conclusions

Taken together, serum Lp-PLA2 levels in KD patients were significantly higher than those in healthy and febrile controls. In addition, KD patients with CAAs had substantially higher serum Lp-PLA2 levels in contrast to the ones without CAAs. Moreover, Lp-PLA2 was positively related to ESR, the levels of WBC, ALB, CK-MB, PLT, and DD, and the *Z-*scores of LMCA, RCA, LAD, and LCX, but negatively related with MCHC and MPV in KD group. When Lp-PLA2 is at the crossroads of lipid metabolism and the inflammatory response, it presents pro-inflammation and proatherogenic effects in some inflammation-related cardiovascular diseases. Base on above findings and evidence, we might conclude that Lp-PLA2 is related to KD and the formation of CAAs. Furthermore, Lp-PLA2 showed superior diagnostic performance for distinguishing KD patients from healthy or febrile controls, and might serve as a potential biomarker for predicting the occurrence of CAAs in KD. These findings might facilitate the early diagnosis and management of KD.

## Study Limitations

There are some limitations in our study. Firstly, the sample size was small, and increasing our sample size could help our results become more convincing. Secondly, whether high levels of serum Lp-PLA2 were the results or the causes of KD was not clear. Finally, the potential mechanisms that Lp-PLA2 may involve in KD and CAA were required to explore. Taken together, future studies are required to further confirm and validate our findings.

## Data Availability Statement

The raw data supporting the conclusions of this article will be made available by the authors, without undue reservation.

## Ethics Statement

The studies involving human participants were reviewed and approved by Ethics Committee of Children's Hospital, Chongqing Medical University. Written informed consent to participate in this study was provided by the participants' legal guardian/next of kin.

## Author Contributions

ZC conceived and designed the study, acquired and analyzed the data, and drafted the paper. HW designed the study, interpreted the data, and revised the manuscript. JZ collected the blood samples. QY critically reviewed and revised the manuscript for important intellectual content and approved its final version. All authors approved the submission of the manuscript in its current form.

## Funding

This work is funded by National Natural Science Foundation of China (No. 81270412).

## Conflict of Interest

The authors declare that the research was conducted in the absence of any commercial or financial relationships that could be construed as a potential conflict of interest.

## Publisher's Note

All claims expressed in this article are solely those of the authors and do not necessarily represent those of their affiliated organizations, or those of the publisher, the editors and the reviewers. Any product that may be evaluated in this article, or claim that may be made by its manufacturer, is not guaranteed or endorsed by the publisher.

## Abbreviations

ALB: albumin
AUC: area under the curve
CAA: coronary artery aneurysm
CK-MB: creatine kinase-MB
DD: D-dimer
ESR: erythrocyte sedimentation rate
FC: febrile control
HC: healthy control
HDL: high-density lipoprotein
IVIG: Intravenous immunoglobulin
KD: Kawasaki disease
LAD: left anterior descending coronary artery
LCX: left circumflex coronary artery
LDL: low-density lipoprotein
LMCA: left main coronary artery
Lp-PLA2: lipoprotein-associated phospholipase A2
MCHC: mean corpuscular hemoglobin concentration
NCAA: non-CAA
PLT: platelet
RCA: right coronary artery
ROC: receiver operating characteristic
WBC: white blood cell.
