# Supplementary material for: The Relationship Between Lipoprotein-Associated Phospholipase-A2 and Coronary Artery Aneurysm in Children With Kawasaki Disease
Source: Front Pediatr. 2022 Mar 31;10:854079. doi: 10.3389/fped.2022.854079 (PMC9008257; doi:10.3389/fped.2022.854079)
Supplement: Supplementary file 1 [file Table_1.pdf]

Table S1. General characteristics of all recruited subjects

	KD (n=71)	HC (n=63)	FC (n=51)	*p*-value
Age(months)	29.44±22.52	30.33±11.59	28.05±14.68	0.783
Gender(male/female)	46/25	41/22	29/22	0.4106

	KD-CAA (n=33)	KD-NCAA	*p*-value
Age(months)	20.31±15.99	37.38±24.48	0.001*
Gender(male/female)	23/10	23/15	0.467

Notes: KD: Kawasaki disease; HCs: healthy controls; FCs: febrile controls; CAA: coronary artery aneurysm; NCAA: non-CAA .
