# Supplementary material for: The Relationship Between Lipoprotein-Associated Phospholipase-A2 and Coronary Artery Aneurysm in Children With Kawasaki Disease
Source: Front Pediatr. 2022 Mar 31;10:854079. doi: 10.3389/fped.2022.854079 (PMC9008257; doi:10.3389/fped.2022.854079)
Supplement: Supplementary file 2 [file Table_2.pdf]

Table S2. The blood coagulation parameters in KD-CAA and KD-NCAA groups

	KD-CAAs（n=33）	KD-NCAAs（n=38）	*p*-value
PLT (10^3/μl)	456.30±179.40	369.90±110.10	0.0181*
MPV(fl)	9.65±0.86	9.93±0.98	0.2588
PDW(fl)	10.46±1.58	10.98±1.95	0.2720
PT (s)	12.75±1.70	12.41±0.74	0.2950
APTT (s)	30.29±3.39	29.91±4.23	0.6902
FIB (g/l)	5.39±1.68	6.001±1.28	0.1010
TT (s)	15.28±1.55	14.73±0.626	0.0602
DD (mg/l)	1.07(0.77-1.90)	1.18(0.61-2.29)	0.9892

*Note: Lp-PLA2: lipoprotein-associated phospholipase A2; KD: Kawasaki disease; CAAs: coronary artery aneurysms; NCAAs: non-CAAs; PLT: platelet counts; MPV: mean platelet volume; PDW: platelet distribution width; PT: prothrombin time; APTT: activated partial thromboplastin time ; FIB: fibrinogen; TT: thrombin time; DD: D-dimer; *, P<0.05.*
