# Supplementary material for: The Relationship Between Lipoprotein-Associated Phospholipase-A2 and Coronary Artery Aneurysm in Children With Kawasaki Disease
Source: Front Pediatr. 2022 Mar 31;10:854079. doi: 10.3389/fped.2022.854079 (PMC9008257; doi:10.3389/fped.2022.854079)
Supplement: Supplementary file 3 [file Table_3.pdf]

Table S3. Z value in KD-CAA and KD-NCAA groups

	KD-CAAs（n=33）	KD-NCAAs（n=38）	*p*-value
z-value of LMCA	3.27±1.51	1.43±0.58	<0.0001*
z-value of RCA	2.61(1.79-3.51)	0.88(0.63-1.46)	<0.0001*
z-value of LAD	1.47(0.57-2.49)	0.45(-0.17-0.78)	0.0006*
z-value of LCX	3.70±1.31	1.55±0.52	<0.0001*

*Note: Lp-PLA2: lipoprotein-associated phospholipase A2; KD: Kawasaki disease; LMCA: left main coronary artery; RCA: right coronary artery; LAD: left anterior descending coronary artery; LCX: left circumflex coronary artery; *, P<0.05.*
